# Health behaviour change among UK adults during the pandemic: findings from the COVID-19 cancer attitudes and behaviours study

**DOI:** 10.1186/s12889-022-13870-x

**Published:** 2022-07-28

**Authors:** Philip Anyanwu, Yvonne Moriarty, Grace McCutchan, Detelina Grozeva, Mark Goddard, Victoria Whitelock, Rebecca Cannings-John, Harriet Quinn-Scoggins, Jacqueline Hughes, Ardiana Gjini, Julie Hepburn, Kirstie Osborne, Michael Robling, Julia Townson, Jo Waller, Katriina L. Whitaker, Jamie Brown, Kate Brain, Graham Moore

**Affiliations:** 1 Centre for Medical Education, School of Medicine, Cardiff University, Cardiff, UK; 2 Division of Population Medicine, School of Medicine, Cardiff University, Cardiff, UK; 3 Centre for Trials Research, Cardiff University, Cardiff, UK; 4 Cancer Intelligence, Cancer Research UK, London, UK; 5 PRIME Centre Wales, Division of Population Medicine, School of Medicine, Cardiff University, Cardiff, UK; 6 Public Health Wales, Cardiff, UK; 7 Cardiff University, Cardiff, UK; 8 Public Involvement Community, Health and Care Research Wales Support Centre, Cardiff, UK; 9 DECIPHer (Centre for Development, Evaluation, Complexity and Implementation in Public Health Improvement), School of Social Sciences, Cardiff University, Cardiff, UK; 10 School of Cancer and Pharmaceutical Sciences, King’s College London, London, UK; 11 School of Health Sciences, University of Surrey, Guildford, UK; 12 Department of Behavioural Science and Health, University College London, London, UK

**Keywords:** Coronavirus disease 2019 (COVID-19), Lockdown, Health behaviour change, Attitudes

## Abstract

**Background:**

COVID-19 related lockdowns may have affected engagement in health behaviours among the UK adult population. This prospective observational study assessed socio-demographic patterning in attempts to change and maintain a range of health behaviours and changes between two time points during the pandemic.

**Methods:**

Adults aged 18 years and over (*n* = 4,978) were recruited using Dynata (an online market research platform) and the HealthWise Wales platform, supplemented through social media advertising. Online surveys were conducted in August/September 2020 when lockdown restrictions eased in the UK following the first major UK lockdown (survey phase 1) and in February/March 2021 during a further national lockdown (survey phase 2). Measures derived from the Cancer Awareness Measure included self-reported attempts to reduce alcohol consumption, increase fruit/vegetable consumption, increase physical activity, lose weight and reduce/stop smoking. Multivariable logistic regressions were used to assess individual health behaviour change attempts over time, adjusted for age, sex, ethnicity, employment and education.

**Results:**

Around half of participants in survey phase 1 reported trying to increase physical activity (*n* = 2607, 52.4%), increase fruit/vegetables (*n* = 2445, 49.1%) and lose weight (*n* = 2413, 48.5%), with 19.0% (*n* = 948) trying to reduce alcohol consumption among people who drink. Among the 738 participants who smoked, 51.5% (*n* = 380) were trying to reduce and 27.4% (*n* = 202) to stop smoking completely. Most behaviour change attempts were more common among women, younger adults and minority ethnic group participants. Efforts to reduce smoking (aOR: 0.98, 95% CI: 0.82–1.17) and stop smoking (aOR: 0.98, 95% CI: 0.80–1.20) did not differ significantly in phase 2 compared to phase 1. Similarly, changes over time in attempts to improve other health behaviours were not statistically significant: physical activity (aOR: 1.07; 95% CI: 0.99–1.16); weight loss (aOR: 0.95; 95% CI: 0.90–1.00); fruit/vegetable intake (aOR: 0.98, 95% CI: 0.91–1.06) and alcohol use (aOR: 1.32, 95% CI: 0.92–1.91).

**Conclusion:**

A substantial proportion of participants reported attempts to change health behaviours in the initial survey phase. However, the lack of change observed over time indicated that overall motivation to engage in healthy behaviours was sustained among the UK adult population, from a period shortly after the first lockdown toward the end of the second prolonged lockdown.

**Supplementary Information:**

The online version contains supplementary material available at 10.1186/s12889-022-13870-x.

## Introduction

The importance of maintaining healthy behaviours to protect against chronic diseases such as cancer, heart disease and diabetes is well documented [1]. During the COVID-19 pandemic, the benefits of adopting healthy behaviours were reiterated due to associations between the severity of COVID-19 and tobacco smoking [2, 3] obesity [4] and alcohol dependence [5]. When the World Health Organisation declared COVID-19 a pandemic on 11^th^ March 2020, over 100 countries around the world went into partial or full lockdown to control the spread of the virus. While lockdown rules varied by country, many countries initially imposed strict ‘Stay at Home’ protection orders [6–9]. The UK government, for example, mandated people to stay at home unless leaving for essential reasons, including food, medicine, medical help, exercise and travel to/from work if they could not work from home. These measures resulted in more people staying at home for longer, which may have influenced engagement in health behaviours. Strict ‘Stay at Home’ orders remained for several months, with gradual easing from late spring/early summer of 2020, with some variation between UK nations. COVID-19 cases remained relatively low through the summer of 2020, but rose rapidly again from the Autumn onwards, leading to additional short lockdowns in Autumn 2020 and another prolonged period of lockdown from December 2020 to March 2021.

Recent systematic reviews report a seemingly negative impact of the pandemic on health behaviours, with greater sedentary time [10, 11], increased snacking [12] and decreased physical activity [11, 13] associated with weight gain [12, 14]. However, over three-quarters of studies included in these reviews used a cross-sectional design, limiting the inferences that can be drawn regarding health behaviour change due to the pandemic [10–14]. In one systematic review of cross-sectional studies, most participants reported no change in alcohol consumption during the pandemic [12]. However, in those who did report increased alcohol use, between 10.4% and 38.5% of participants reported increased alcohol consumption during the COVID-19 lockdowns [12]. Empirical studies have found no change in smoking behaviours for most participants during the pandemic [15–17], although significant increases in quit attempts and cessation have been observed [18, 19]. Increased smoking prevalence and increased quit attempts were observed among younger adults [9] and increased high-risk drinking among all adults [10]. There may be variation between countries in relation to health behaviour changes; for example, alcohol purchasing behaviour during the pandemic increased in North England but reduced in Wales and Scotland [20].

Evidence points to the unequal burden of COVID-19, with higher infection rates and worse outcomes among people with smoking histories, people from Black, Asian and minority ethnic backgrounds and those in lower socioeconomic groups [21, 22]. Significant disparities in engagement with health behaviours have also been observed, including a possible polarising effect of alcohol consumption [23] higher rates of high-risk drinking among women and people from lower socioeconomic groups [10], and decreased levels of physical activity among lower socioeconomic groups [11], with more barriers to accessing healthy food among low-income groups during the pandemic [24].

**Fig. 1:**
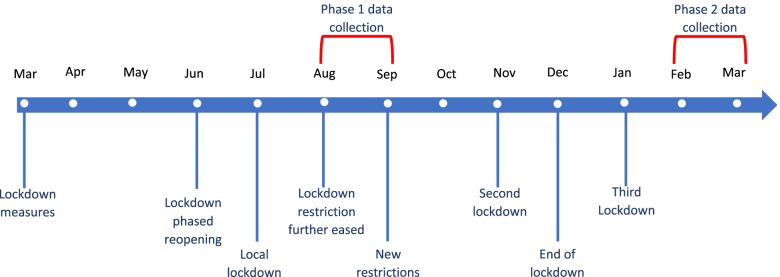
Timeline of UK COVID-19 pandemic restrictions and data collection

We report prospective data from the COVID-19 Cancer Attitudes and Behaviours study (CABs) [25, 26] and the COVID-19 Cancer Awareness Measure regarding efforts among the UK adult population to change self-reported health behaviours, including smoking, alcohol consumption, fruit and vegetable intake, physical activity and weight loss during the COVID-19 pandemic. Phase 1 survey data were collected in late summer of 2020 when COVID-19 infection control measures were eased, and people’s motivation for behaviour change may have been higher than at earlier stages of the pandemic and prior to the escalation of the second pandemic wave. Phase 2 data were collected in early spring of 2021 toward the end of two further prolonged periods of national lockdown following the emergence of the Alpha variant in Autumn 2020 (see Fig. 1). We therefore examined the prevalence and demographic patterning of behaviour change efforts during a period of relative quiescence, and the extent to which these efforts changed through the second pandemic wave.

## Methods

### Study design

The study used prospective observational data derived from a broader study of cancer symptom help-seeking and screening behaviour in the UK adult population during COVID-19. The study protocol and analysis plans were pre‐registered on Open Science Framework [26]. Findings are reported in accordance with the STROBE guidelines for surveys and observational studies. [27, 28].

### Participants and data

Participants were English-speaking adults aged 18 years and over, resident in the UK and recruited using Cancer Research UK’s online panel provider (Dynata), HealthWise Wales (a national register of ‘research ready’ participants) [29] and social media platforms (Facebook and Twitter), then followed up over time. Representation of people who smoke and those from ethnic minority and lower socioeconomic groups was increased by using targeted advertising and placing quotas on sample size relative to UK population statistics. Two online surveys were conducted in parallel: CABs (participants recruited via HealthWise Wales and social media) and the COVID-19 Cancer Awareness Measure (COVID-CAM; participants recruited via Dynata). Survey data were pooled where appropriate [25, 26]. Prospective data were collected in two phases from the same participants during August–September 2020 (phase 1) and February–March 2021 (phase 2). Sampling and recruitment methods are reported in detail elsewhere [25].

### Measures

Measures of behaviour change attempts were derived from the Cancer Awareness Measure 2019 [30]. Participants were asked “Are you currently trying to do any of the following?” for each of the following behaviours: “Reduce the amount you smoke”; “Stop smoking completely”; “Reduce the amount of alcohol you drink”; “Increase the amount of fruit and vegetables you eat”; “Increase the amount of physical activity you do”, and “Lose weight”. Response options were “Yes”, “No”, “Maybe”, “Prefer not to say”, and “This is not applicable to me”.

Smoking status was ascertained by asking participants “Which of the following best describes you?” with response options “I have never smoked”, “I used to smoke but have given up”, “I smoke but not every day”, “I smoke every day”, “Other” and “Prefer not to say”. Demographic variables included age, sex, ethnicity, employment and educational qualification [25].

### Variables

Outcome variables were binary measures of smoking status and attempts to change five behaviours: smoking (i.e. attempts to reduce smoking and attempts to stop smoking), alcohol consumption, fruit and vegetable intake, physical activity and weight loss. The predictor variable was a time variable with a binary measure to indicate the first (August–September 2020) and second (February–March 2021) survey phases.

### Handling missing data

The rate of missing values in the outcome variables ranges from 0 to 8% (see Supplementary Table 1). We examined whether missingness in the health behaviour variables was patterned by participants’ characteristics (gender, age, ethnicity, country, occupation, and educational qualification). Although missing values in the outcome variables were mostly not patterned by participants’ demography, we found differences in the odds of missing data in some health behaviour variables according to educational qualification (those with other qualifications or no qualification were more likely to have missing value for fruit and vegetable intake, physical activity and alcohol use than those with a degree) and occupation (those unemployed and retired were more likely to have missing data for alcohol use and weight loss compared to those employed).

In order to minimise bias, missing values were handled using multiple imputation, which ensures that all observed values in a dataset with some systematic differences between the missing and observed values are retained [31, 32]. Outcome and demographic variables informed the multiple imputations model. Results were averaged across ten imputed datasets, and the sample characteristics of the imputed sample did not differ considerably from those of the fully observed person-years.

### Statistical analysis

Analyses were conducted using Stata (version 17). Descriptive statistics were used to analyse the proportion of participants (overall and by demographic variables) engaging in each health behaviour attempt at each time point. Univariable and multivariable logistic regression analyses were carried out to assess differences in attempts to change each behaviour (smoking, alcohol, fruit/vegetable intake, physical activity and weight loss) between the two phases. Results for all models are presented as odds ratios with 95% confidence intervals. Participants’ age, sex, ethnicity, UK country, employment status and educational qualification were included as hypothesised confounders. Complete case analyses were conducted, with sensitivity analysis using the imputed dataset to account for potential attrition bias.

Subgroup analyses were pre-specified and conducted by introducing interaction terms between the predictor (time) and demographic variables (sex, ethnicity, employment and education).

## Results

### Sample characteristics

Participants (*n* = 4,978) were adults aged 18 years and over from the four UK countries (37.9% from England, 32.4% from Wales, 4.0% from Scotland, 0.70% from Northern Ireland) who completed the surveys at both time points. Over half of the participants were males (53.5%) and the majority identified as White (91.8%). The majority of participants were aged below 65 years (61.4%) and employed (47.2%). For educational attainment, 40% of participants had a degree or higher. In phase 1, around half of participants reported trying to increase physical activity (*n* = 2607, 52.4%), increase fruit/vegetable intake (*n* = 2445, 49.1%) and lose weight (*n* = 2413, 48.5%), with 19.0% (*n* = 948) trying to reduce alcohol. Among the 738 participants who smoked, 51.5% (*n* = 380) were trying to reduce and 27.4% (*n* = 202) to stop smoking completely.

### Behaviour change attempts

In Phase 1, across all outcomes other than attempts to reduce or stop smoking, there were more behaviour change efforts among women and for the two younger age groups, with a tendency for behaviour change efforts to become less common with increased age (Table 1). In Phase 1, retired people consistently reported fewer behaviour change efforts across all outcomes, with smaller and less consistent patterning in differences between employed and unemployed respondents. For all behaviour change attempts, respondents from minority ethnic groups reported more efforts to change in Phase 1. The evidence of consistent socio-demographic patterning in behaviour change attempts in Phase 1 (i.e. sex, age and ethnicity) remained largely unchanged in Phase 2.

**Table 1:** Distribution of health behaviours by covariates across the two study phases, *N* = 4,978

		**Smokers** n(%)		**Attempts to reduce smoking**^**a**^ n(%)		**Attempts to stop smoking**^**a**^ n(%)		**Attempts to increase fruit intake** n(%)		**Attempts to increase physical activity** n(%)		**Attempts to reduce alcohol use** n(%)		**Attempts to lose weight** n(%)	
		Phase 1	Phase 2	Phase 1	Phase 2	Phase 1	Phase 2	Phase 1	Phase 2	Phase 1	Phase 2	Phase 1	Phase 2	Phase 1	Phase 2
**Total**		738 (14.8)	728 (14.6)	380 (51.5)	352 (48.3)	202 (27.4)	169 (23.2)	2445 (49.1)	2417 (48.6)	2607 (52.4)	2682 (53.9)	948 (19.0)	944 (19.0)	2413 (48.5)	2295 (46.1)
**Sex**	Male	386 (14.7)	388 (14.8)	200 (51.8)	183 (47.2)	98 (25.4)	76 (19.6)	1156 (56.0)	1129 (55.4)	1235 (59.2)	1295 (61.7)	509 (28.3)	511 (28.4)	1113 (53.1)	1075 (51.5)
	Female	351 (15.4)	339 (14.9)	179 (51.0)	168 (49.5)	103 (29.3)	93 (27.4)	1286 (69.3)	1285 (70.0)	1367 (73.8)	1383 (74.6)	438 (33.4)	433 (32.8)	1296 (68.8)	1217 (66.3)
**Age**	75 and above	18 (4.6)	15 (3.8)	6 (33.3)	5 (33.3)	1 (5.5)	2 (13.3)	132 (48.5)	131 (48.5)	150 (54.5)	155 (56.0)	32 (14.7)	39 (18.1)	147 (51.2)	141 (49.1)
	65 to 74	113 (7.6)	114 (7.6)	53 (46.9)	55 (48.2)	24 (21.2)	22 (19.3)	592 (52.8)	620 (56.1)	662 (58.1)	728 (63.8)	223 (24.4)	210 (23.2)	685 (58.1)	652 (56.9)
	55 to 64	140 (14.1)	126 (12.7)	74 (52.8)	64 (50.8)	39 (27.8)	29 (20.7)	491 (60.5)	474 (59.8)	541 (66.3)	548 (66.9)	199 (30.9)	211 (32.0)	509 (62.8)	488 (60.3)
	45 to 54	162 (19.7)	165 (20.0)	91 (56.2)	72 (43.6)	43 (26.5)	38 (23.0)	478 (70.2)	455 (68.1)	486 (71.2)	468 (68.9)	191 (35.5)	180 (33.5)	426 (62.3)	396 (60.1)
	35 to 44	148 (23.8)	144 (23.1)	78 (52.7)	74 (51.4)	48 (32.4)	35 (24.3)	360 (69.5)	351 (67.5)	380 (73.4)	390 (73.9)	158 (39.3)	167 (39.0)	332 (64.0)	315 (61.4)
	25 to 34	120 (28.2)	124 (29.2)	57 (47.5)	65 (52.4)	36 (30.0)	34 (27.4)	291 (78.6)	279 (74.4)	286 (77.9)	285 (76.6)	107 (37.8)	102 (37.8)	233 (64.5)	223 (60.6)
	18 to 24	31 (25.8)	33 (27.3)	20 (64.5)	15 (45.4)	10 (32.2)	8 (24.2)	79 (73.1)	85 (73.9)	79 (72.5)	81 (77.1)	26 (30.6)	29 (34.5)	57 (51.4)	56 (52.8)
**Ethnicity**	White	668 (14.8)	648 (14.3)	338 (50.6)	309 (47.7)	176 (26.3)	142 (21.9)	2196 (61.3)	2178 (61.4)	2350 (65.2)	2432 (66.9)	850 (29.4)	853 (29.3)	2199 (60.2)	2096 (58.2)
	Ethnic minorities	69 (17.8)	80 (20.6)	42 (60.9)	43 (53.7)	26 (37.7)	26 (32.5)	246 (74.1)	236 (72.4)	254 (76.3)	247 (76.7)	97 (43.9)	90 (43.1)	211 (63.0)	196 (60.1)
**Country**	England	314 (16.8)	311 (16.6)	155 (49.4)	153 (49.2)	81 (25.8)	79 (25.4)	973 (61.7)	934 (60.1)	1001 (64.7)	1014 (66.3)	381 (30.3)	387 (30.0)	915 (58.9)	884 (58.4)
	Scotland	34 (17.3)	38 (19.2)	14 (41.2)	15 (39.5)	5 (14.7)	6 (15.8)	110 (68.3)	117 (66.9)	106 (65.4)	111 (65.7)	38 (30.4)	45 (32.1)	101 (58.7)	91 (54.2)
	Northern Ireland	5 (13.5)	5 (13.5)	3 (60.0)	3 (60.0)	0 (0.0)	1 (20.0)	16 (55.2)	19 (61.3)	22 (66.7)	21 (61.8)	9 (36.0)	6 (25.0)	18 (62.1)	18 (56.3)
	Wales	131 (8.3)	127 (8.0)	67 (51.1)	58 (45.7)	40 (30.5)	32 (25.2)	671 (60.3)	665 (61.4)	788 (67.1)	838 (69.3)	231 (26.5)	216 (25.9)	782 (64.6)	760 (63.5)
**Employment**	Employed	457 (19.8)	449 (19.4)	259 (56.7)	230 (51.2)	159 (34.8)	124 (27.6)	1323 (68.7)	1284 (67.2)	1420 (72.9)	1403 (71.8)	578 (36.3)	571 (35.8)	1221 (62.6)	1154 (60.7)
	Retired	141 (7.1)	136 (6.8)	59 (41.8)	61 (44.8)	22 (15.6)	20 (14.7)	763 (51.0)	782 (53.5)	866 (56.9)	956 (62.9)	269 (22.3)	266 (22.0)	890 (57.5)	851 (55.3)
	Unemployed	137 (23.1)	139 (23.4)	60 (43.8)	58 (41.7)	21 (15.3)	24 (17.3)	351 (72.2)	344 (69.6)	311 (67.5)	312 (65.8)	97 (31.7)	103 (33.0)	294 (61.3)	284 (59.3)
**Education**	Degree or higher	241 (12.4)	240 (12.4)	124 (51.4)	116 (48.3)	76 (31.5)	55 (22.9)	945 (59.8)	927 (59.7)	1099 (67.9)	1122 (70.5)	400 (30.9)	397 (31.1)	980 (61.8)	921 (58.7)
	No qualification	48 (16.9)	46 (16.1)	17 (35.4)	22 (47.8)	7 (14.6)	6 (13.0)	130 (63.1)	138 (64.8)	119 (56.1)	117 (55.5)	35 (24.0)	39 (23.9)	129 (56.3)	119 (54.3)
	Other qualifications	442 (16.6)	433 (16.3)	237 (60.5)	210 (48.5)	117 (26.5)	105 (24.2)	1351 (64.2)	1332 (63.9)	1366 (65.7)	11,420 (66.7)	400 (30.9)	505 (30.3)	1284 (59.8)	1236 (58.5)

^a^Proportion of those who reported that they currently smoke

### Logistic regression analyses

Univariable models with each health behaviour regressed against the covariates are reported in Tables 2 and 3.

**Table 2:** Univariable models for smoking behaviours

Predictors		Smoking status			Reducing smoking			Stop smoking		
		Odds Ratio	95% Confidence Intervals		Odds Ratio	95% Confidence Intervals		Odds Ratio	95% Confidence Intervals	
			Lower	Upper		Lower	Upper		Lower	Upper
Phase (ref: phase 1)		0.97	0.93	1.02	0.98	0.85	1.12	0.89	0.72	1.04
Sex (ref: female)		0.97	0.83	1.12	0.80	0.62	1.03	0.67	0.51	0.90
Age in years (ref: 18 to 24)	75 and above	0.13	0.07	0.23	0.19	0.07	0.50	0.10	0.02	0.48
	65 to 74	0.23	0.15	0.35	0.46	0.23	0.93	0.40	0.19	0.83
	55 to 64	0.43	0.29	0.65	0.73	0.36	1.46	0.64	0.31	1.30
	45 to 54	0.70	0.47	1.04	0.93	0.46	1.86	0.83	0.41	1.70
	35 to 44	0.85	0.57	1.28	1.16	0.57	2.35	1.03	0.51	2.11
	25 to 34	1.14	0.75	1.73	0.94	0.46	1.90	1.03	0.50	2.12
Ethnic minorities (ref: White)		1.41	1.10	1.80	1.98	1.27	3.09	2.25	1.43	3.52
Country (ref: England)	Northern Ireland	0.78	0.30	2.02	0.97	0.16	5.90	0.44	0.40	4.25
	Scotland	1.11	0.77	1.60	0.71	0.37	1.37	0.43	0.19	1.02
	Wales	0.45	0.36	0.55	0.32	0.23	0.45	0.41	0.28	0.60
Employment (ref: employed)	Retired	0.31	0.25	0.37	0.36	0.26	0.50	0.24	0.16	0.36
	Unemployed	1.23	1.00	1.52	0.76	0.53	1.10	0.47	0.31	0.71
Education (ref: Degree or higher)	No qualification	1.42	1.02	1.96	0.82	0.47	1.41	0.53	0.25	1.12
	Other qualifications	1.39	1.18	1.63	1.43	1.10	1.88	1.14	0.85	1.54

**Table 3:** Univariable models for all other health behaviours

Predictors		Increased physical activity			Reduced alcohol use			Weight loss			Increased fruit intake		
		Odds Ratio	95% Confidence Intervals		Odds Ratio	Odds Ratio		Odds Ratio	Odds Ratio		Odds Ratio	95% Confidence Intervals	
			Lower	Upper		Lower	Upper		Lower	Upper		Lower	Upper
Phase (ref: phase 1)		1.05	0.99	1.12	0.98	0.91	1.05	0.92	0.88	0.96	1.00	0.94	1.06
Sex (ref: female)		0.55	0.49	0.61	0.83	0.73	0.94	0.55	0.49	0.61	0.56	0.50	0.62
Age in years (ref: 18 to 24)	75 and above	0.43	0.28	0.65	0.40	0.25	0.65	0.92	0.58	1.29	0.35	0.23	0.52
	65 to 74	0.52	0.36	0.76	0.65	0.44	0.96	1.24	0.82	1.67	0.42	0.29	0.61
	55 to 64	0.66	0.45	0.97	0.96	0.65	1.43	1.47	0.96	1.99	0.54	0.37	0.78
	45 to 54	0.79	0.53	1.17	1.05	0.70	1.56	1.39	0.90	1.88	0.78	0.53	1.14
	35 to 44	0.94	0.63	1.40	1.30	0.87	1.95	1.53	0.99	2.09	0.78	0.53	1.15
	25 to 34	1.10	0.73	1.68	1.23	0.81	1.87	1.45	0.93	2.01	1.12	0.75	1.68
Ethnic minorities (ref: White)		1.63	1.31	2.03	1.77	1.40	2.24	1.07	0.87	1.31	1.68	1.36	2.07
Country (ref: England)	Northern Ireland	0.96	0.51	1.83	1.01	0.49	2.06	1.04	0.53	2.02	0.92	0.47	1.80
	Scotland	0.98	0.73	1.30	1.08	0.78	1.51	0.91	0.68	1.21	1.39	1.05	1.83
	Wales	1.13	0.98	1.29	0.84	0.71	0.99	1.22	1.06	1.40	1.00	0.88	1.15
Employment (ref: employed)	Retired	0.58	0.52	0.66	0.51	0.44	0.59	0.81	0.72	0.92	0.53	0.47	0.59
	Unemployed	0.77	0.64	0.92	0.82	0.66	1.03	0.94	0.79	1.13	1.13	0.95	1.36
Education (ref: Degree or higher)	No qualification	0.56	0.44	0.71	0.73	0.53	1.02	0.87	0.67	1.22	1.22	0.96	1.56
	Other qualifications	0.87	0.77	0.98	0.97	0.85	1.11	0.94	0.84	1.06	1.20	1.07	1.34

As depicted in Table 2, for the model without adjustment, the likelihood of smoking did not differ in Phase 2 compared to Phase 1 (OR: 0.97, 95% CI: 0.93–1.02). The OR remained consistent after extending the model to adjust for confounders, including sex, age, ethnicity, UK country, employment and education (aOR: 0.98, 95% CI: 0.93–1.04). Efforts to reduce and stop smoking did not differ substantially over time for the adjusted and unadjusted models (reducing smoking: aOR: 0.98, 95% CI: 0.82–1.17; stop smoking: aOR: 0.98, 95% CI: 0.80–1.20).

There were no substantial differences in attempts to improve other health behaviours over time: increased physical activity (aOR: 1.07; 95% CI: 0.99–1.16); weight loss (aOR: 0.95; 95% CI: 0.90–1.00); increased fruit intake (aOR: 0.98, 95% CI: 0.91–1.06) and reduced alcohol use (aOR: 1.32, 95% CI: 0.92–1.91) (see Table 3 for unadjusted estimates).

### Subgroup analyses

We introduced interaction terms to the models to investigate whether any difference in behaviours between the first and second phases was moderated by sex, ethnicity, employment and education. Consistent with descriptive data, which indicated similar demographic patterning in behaviour change attempts at both phases, subgroup analyses for all health behaviours showed no significant interaction effect between the time variable and the moderators.

### Sensitivity analyses

The results of complete case analyses were broadly consistent with those using the imputed set (see Supplementary Table 2), with some differences in the results for increased physical activity and weight loss. Although the ORs for the predictor variable (indicating phase 1 or 2) on both outcomes were similar in the analyses with complete cases and imputed datasets, they were significant in the imputed set analyses for increased physical activity (aOR: 1.09; 95% CI: 1.01–1.17) and weight loss (aOR: 0.92; 95% CI: 0.87–0.99), meaning there were more physical activity attempts but less attempts to lose weight at Phase 2 compared to Phase 1.

## Discussion

This prospective study assessed attempts to change a range of health behaviours among UK adults during the COVID-19 pandemic. Around half of the participants were attempting to change health behaviours, including smoking, during a relatively dormant phase of the pandemic in late summer 2020, with no observable changes by early spring 2021 when two further national lockdowns had taken place. When continued infection control measures might have compromised people’s health motivation during the pandemic's life course, we observed consistency in willingness to engage in health behaviours.

Our findings indicating the absence of significant change over time in smoking behaviours are similar to those of other studies during the pandemic, from a period of relative normality after the first prolonged lockdown through the end of the second major wave of infection and lockdown in the UK nations [15, 33]. Although the timeline of our study differs from that of Naughton et al. [33] (their follow-up period coincided with our first survey phase), our study mirrors their findings related to smoking behaviour; they reported no difference in smoking status and smoking frequency during the UK COVID-19 lockdown. Similar to our study, Niedzwiedz et al. [34] did not observe any differential impact of the pandemic lockdown on smoking across age, sex, ethnicity or socioeconomic subgroups. However, we noted that the proportion of participants in ethnic minority groups who self-reported as smokers in our study increased by 2.8% in the second compared to the first study phase.

In contrast with systematic reviews that have described reduced physical activity [11, 13] in the UK population during the pandemic, we observed a slight overall increase in self-reported attempts to increase physical activity. Attempts to lose weight appeared to diminish over time in our study, in line with previous studies that reported increased behaviours related to weight gain [10–12]. Changes in fruit/vegetable intake and alcohol consumption were not observed [12].

The current study findings suggest that health behaviour change attempts were generally higher among younger adults and people in ethnic minority groups. A similar trend in behaviour change attempts by age during the COVID-19 pandemic was reported in another UK study where older-aged cohorts were less likely to report changes in exercise, alcohol, and fruit and vegetable consumption than the younger cohorts [35]. However, their finding on health behaviour change by ethnicity differed from our study. Contrary to our findings, they reported lower fruit and vegetable consumption and lower exercise rates among ethnic minorities than white participants.

Although a higher risk of severe outcomes of COVID-19 is seen in men than women [36, 37] attempts towards health behaviour change were overall higher in women in both phases of our study compared to men. Our univariable models with sex showed that behaviour change attempts were significantly lower in men than in women for five out of the seven health behaviours tested in our study. A similar association was reported in a Taiwanese study where females had higher odds of adopting health-protective behaviours during the COVID-19 pandemic than men [38]. In the US, older men were reported to have implemented the fewest behaviour changes during the pandemic [39].

### Limitations

Our analyses were strengthened by using a repeated-measures dataset, addressing the gap in evidence from cross-sectional studies. However, our study has some limitations. A larger proportion of our participants were recruited from a database of individuals interested in health-related research (HealthWise Wales). Therefore, it is possible that participants in our study were more motivated to change health behaviours and that the lack of observed demographic patterning in health behaviour change efforts reflects limited sample variation. Additionally, while we have adjusted for important confounders and effect modifiers, other factors might explain the consistency in health behaviours between both waves. For instance, unmeasured variables such as the living conditions of participants (e.g. whether they lived with other household members who attempted to change their behaviours during the pandemic) and the presence of underlying chronic conditions that increase the risk of COVID-19 complications have been reported to be associated with smoking cessation during the COVID-19 lockdown in other countries [40].

Some of our variables had missing values. The higher rate of missing values among those of lower education and unemployed for some of the health behaviours could lead to underestimation of effect; however, consistency between the results of complete case analysis and sensitivity analysis using an imputed dataset demonstrated a low risk of attrition bias. Finally, self-report measures used in the current study may not accurately reflect actual change in health behaviours.

## Conclusion

A substantial proportion of participants reported attempts to change health behaviours in the initial survey phase. The lack of observed differences in self-reported health behaviours over time suggests an unexpected degree of behavioural consistency at a time when pandemic restrictions might have been expected to worsen motivation. This is encouraging, and suggests that public health messaging could build on the public's willingness to engage in healthy behaviours. In addition, our findings will enable intervention developers to target behavioural messaging to specific demographic groups who are less likely to engage in behaviour change.

## Supplementary Information

**Additional file 1: Supplementary Table 1.** Proportion of missing values per variable. **Supplementary Table 2.** Complete case vs Imputed case analysis.

## Data Availability

The datasets used and/or analysed during the current study available from the corresponding author on reasonable request.
