# Supplementary material for: Health behaviour change among UK adults during the pandemic: findings from the COVID-19 cancer attitudes and behaviours study
Source: BMC Public Health. 2022 Jul 28;22:1437. doi: 10.1186/s12889-022-13870-x (PMC9332100; doi:10.1186/s12889-022-13870-x)
Supplement: Supplementary file 1 — Additional file 1: Supplementary Table 1. Proportion of missing values per variable. Supplementary Table 2. Complete case vs Imputed case analysis. [file 12889_2022_13870_MOESM1_ESM.pdf]

Supplementary Table 1: Proportion of missing values per variable

Variables	% missing
**Smoking status**	0.11
**Stop smoking**	8.01
**Reduced smoking**	7.88
**Daily smoker**	7.75
**Ex-smoker**	4.51
**Reduced alcohol use**	3.39
**Country**	2.27
**Increased fruit intake**	1.97
**Increased physical activity**	1.87
**Weight loss**	1.86
**Age categories**	0.11
**Qualification**	0.08
**House ownership**	0.08
**Occupation**	0.04
**Ethnicity**	0.01
**Sex**	0

Supplementary Table 2: Complete case vs Imputed case analysis

Health behaviours	Complete case (main analyses)			Imputed set (sensitivity analyses)		
	Odds ratio	95% CI		Odds ratio	95% CI	
		Lower	Upper		Lower	Upper
**Smoking status**	0.98	0.93	1.04	0.98	0.93	1.04
**Reducing smoking**	0.98	0.82	1.17	0.95	0.80	1.14
**Stop smoking**	0.98	0.80	1.20	0.88	0.68	1.13
**Increased physical activity**	1.07	0.99	1.16	1.09	1.01	1.17
**Reduced alcohol use**	1.32	0.92	1.91	1.03	0.93	1.15
**Weight loss**	0.95	0.90	1.00	0.92	0.87	0.99
**Increased fruit intake**	0.98	0.91	1.06	1.00	0.93	1.07
